# Spatio-Temporal Variation in Predation by Urban Domestic Cats (*Felis catus*) and the Acceptability of Possible Management Actions in the UK

**DOI:** 10.1371/journal.pone.0049369

**Published:** 2012-11-16

**Authors:** Rebecca L. Thomas, Mark D. E. Fellowes, Philip J. Baker

**Affiliations:** School of Biological Sciences, Harborne Building, University of Reading, Whiteknights, Reading, Berkshire, United Kingdom; Australian Wildlife Conservancy, Australia

## Abstract

Urban domestic cat (*Felis catus*) populations can attain exceedingly high densities and are not limited by natural prey availability. This has generated concerns that they may negatively affect prey populations, leading to calls for management. We enlisted cat-owners to record prey returned home to estimate patterns of predation by free-roaming pets in different localities within the town of Reading, UK and questionnaire surveys were used to quantify attitudes to different possible management strategies. Prey return rates were highly variable: only 20% of cats returned ≥4 dead prey annually. Consequently, approximately 65% of owners received no prey in a given season, but this declined to 22% after eight seasons. The estimated mean predation rate was 18.3 prey cat^−1^ year^−1^ but this varied markedly both spatially and temporally: per capita predation rates declined with increasing cat density. Comparisons with estimates of the density of six common bird prey species indicated that cats killed numbers equivalent to adult density on *c.* 39% of occasions. Population modeling studies suggest that such predation rates could significantly reduce the size of local bird populations for common urban species. Conversely, most urban residents did not consider cat predation to be a significant problem. Collar-mounted anti-predation devices were the only management action acceptable to the majority of urban residents (65%), but were less acceptable to cat-owners because of perceived risks to their pets; only 24% of cats were fitted with such devices. Overall, cat predation did appear to be of sufficient magnitude to affect some prey populations, although further investigation of some key aspects of cat predation is warranted. Management of the predation behavior of urban cat populations in the UK is likely to be challenging and achieving this would require considerable engagement with cat owners.

## Introduction

Owing to its close association with humans, the domestic cat (*Felis catus*) has one of the largest geographical distributions of any terrestrial carnivore [1], [2]. Within this range, feral populations are known to have caused the decline or extirpation of numerous species [3]–[7]. In many developed countries, most individuals are free-roaming pets or semi-feral and receive some or all of their nutrition from humans [1]. Consequently, unlike other predators [8], [9], their abundance is not limited by natural prey availability and densities can be very high, particularly in urban areas (200–2000 km^−2^ [1], [10], [11]). Yet, despite being fed, pet cats do frequently kill wild prey [12]–[26] and, given their high numbers, the cumulative sum of wildlife killed in urban areas could be substantial, even if individual predation rates are low. Consequently, there has been considerable debate surrounding the possible effect of urban cat populations and whether and/or how they should be managed [17], [27]–[39].

Definitive demonstration of the effect (or not) of urban cats on prey populations has proven problematic as their numbers cannot be manipulated as in conventional predator-prey studies [12], [40]–[43]. To date, only two studies have quantified prey responses to altered cat abundance in urban areas, with contrasting results: the first recorded significant declines of several avian species in the urbanised landscape of southern California [24], whereas the second reported little or no effect of banning/collaring cats on the abundance of native mammalian prey in Armadale, Western Australia [35]. These studies highlight that direct comparisons of studies of predation by urban cats are problematic because of national differences in the definition and application of the term “urban”, variation in available prey bases and differences in cat-owning behaviours. For example, the Californian study mentioned above [25] focussed on predation of habitat specialists within remnant fragments of sage-scrub, whereas within many other countries most predation occurs within gardens in the urban matrix itself.

Given the difficulties associated with manipulating cat numbers experimentally, most authors have instead estimated predation rates from prey returned home [12]–[26]. Although relatively common, many of these studies are of limited use for estimating effects on prey populations. For example, those conducted for short periods do not account for intra-annual variation and most have been conducted in seasons where predation is inherently greater [18], [20]–[23], [25], [26]; these would over-estimate predation rates. Similarly, studies investigating the effectiveness of anti-predation devices [18], [22], [23], [25], [26] have tended to recruit cats known to be proficient hunters, leading to over-estimates of the average for the population as a whole. Despite these caveats, those data available indicate that prey return rates in urban areas are highly variable (e.g. 4–36 prey cat^−1^ year^−1^ [12]–[26]). Converting these to predation rates is further complicated by limited information on the proportion of prey killed that are returned home [18], [44], [45] and the survival rates of prey released alive.

Predation rates, however, are only meaningful in the context of prey availability. At present, annual predation rates in relation to prey availability have been estimated in only five major cities in three countries [12]–[17], [19]. Collectively, these studies have indicated that predation rates can be substantial relative to the breeding density and/or productivity of some prey species. However, most have been conducted in cities in Australia and New Zealand [14]–[17], [19] where domestic cats were only introduced approximately 200–400 years ago [2], [46] and prey species evolved in the absence of placental mammalian predators. Patterns of predation have been quantified in just one major urban conurbation in the Northern Hemisphere (Bristol, UK: [12], [13]) where cats have been present for 1000–2000 years [2], [46].

**Table 1:** Estimated cat density in 16 1-km^2^ squares in Reading.

Grid reference	Method^1^	Total houses	Number of houses responding/contacted[No. of houses with cats]		Mean no. of cats percat- owning household [Median]	Total cat density^2^
			Original phase	Follow-up phase		
SU 6672	B	1060	–	50 [11]	1.91 [1]	445 [243]
SU 6773	A	1668	174 [56]	100 [14]	1.54 [1]	409 [303]
SU 6972	A	617	74 [26]	50 [6]	1.78 [1]	271 [215]
SU 7075	A	1058	131 [45]	50 [14]	1.53 [1]	465 [336]
SU 7269	B	1920	–	55 [17]	1.71 [1]	1012 [605]
SU 7271	B	2004	–	50 [12]	1.42 [1]	681 [486]
SU 7274	B	1708	–	50 [14]	1.36 [1]	649 [483]
SU 7276	A	864	60 [20]	100 [21]	1.80 [1]	341 [222]
SU 7370	A	1575	152 [41]	50 [9]	1.48 [1]	440 [321]
SU 7372	A	1414	121 [44]	50 [4]	1.56 [1]	230 [174]
SU 7471	B	1105	–	50 [17]	1.29 [1]	486 [381]
SU 7473	B	1031	–	50 [8]	1.50 [1]	247 [169]
SU 7570	A	2099	168 [76]	50 [9]	1.62 [1]	688 [477]
SU 7572	A	1301	126 [49]	50 [10]	1.56 [1]	443 [317]
SU 7674	B	734	–	95 [24]	1.46 [1]	270 [196]
SU 7773	A	1632	108 [50]	100 [11]	1.49 [1]	325 [248]

1 A = two-tier approach used: households responding to original leaflet survey termed “original phase”; randomly selected householders interviewed face-to-face are designated “follow-up phase”. B = one-sample survey of randomly selected householders.

2 Figures indicate estimated cat density based on mean number of cats per cat-owning household. Figures in square brackets indicate density based on median number of cats per cat-owning household.

Furthermore, predation rates are not substantial for all prey species or in all locations within the same city [12]. At a general level, this inter- and intra-city variation is likely to be associated with differences in the general abundance of major prey groups but also because of the properties of this rather unusual predator-prey system. For example, the spatial distribution and abundance of cats in the UK is known to be affected by housing density [11] and householder characteristics [47], whereas the abundance of individual prey species may increase, decline or peak at intermediate levels of housing density [11], [48]. Further complexity may also arise through differences in owner behaviour (e.g. restrictions on the time(s) of day or night that cats are allowed outside, the fitting of cats with anti-predation devices) and variation in the hunting tendencies of individual cats [14], [15], [49], especially as wild prey are not typically needed for nutritional purposes. In addition, individual cats may also compete with one another as density increases affecting per capita predation rates. As a consequence, effects of free-roaming cats on the population size of individual prey species are likely to be varied and may not necessarily simply reflect cat density [11]. At present, there are very few data on how patterns of predation vary spatially, intra-annually and, in particular, inter-annually within urban areas (but see [12], [17]).

**Figure 1:**
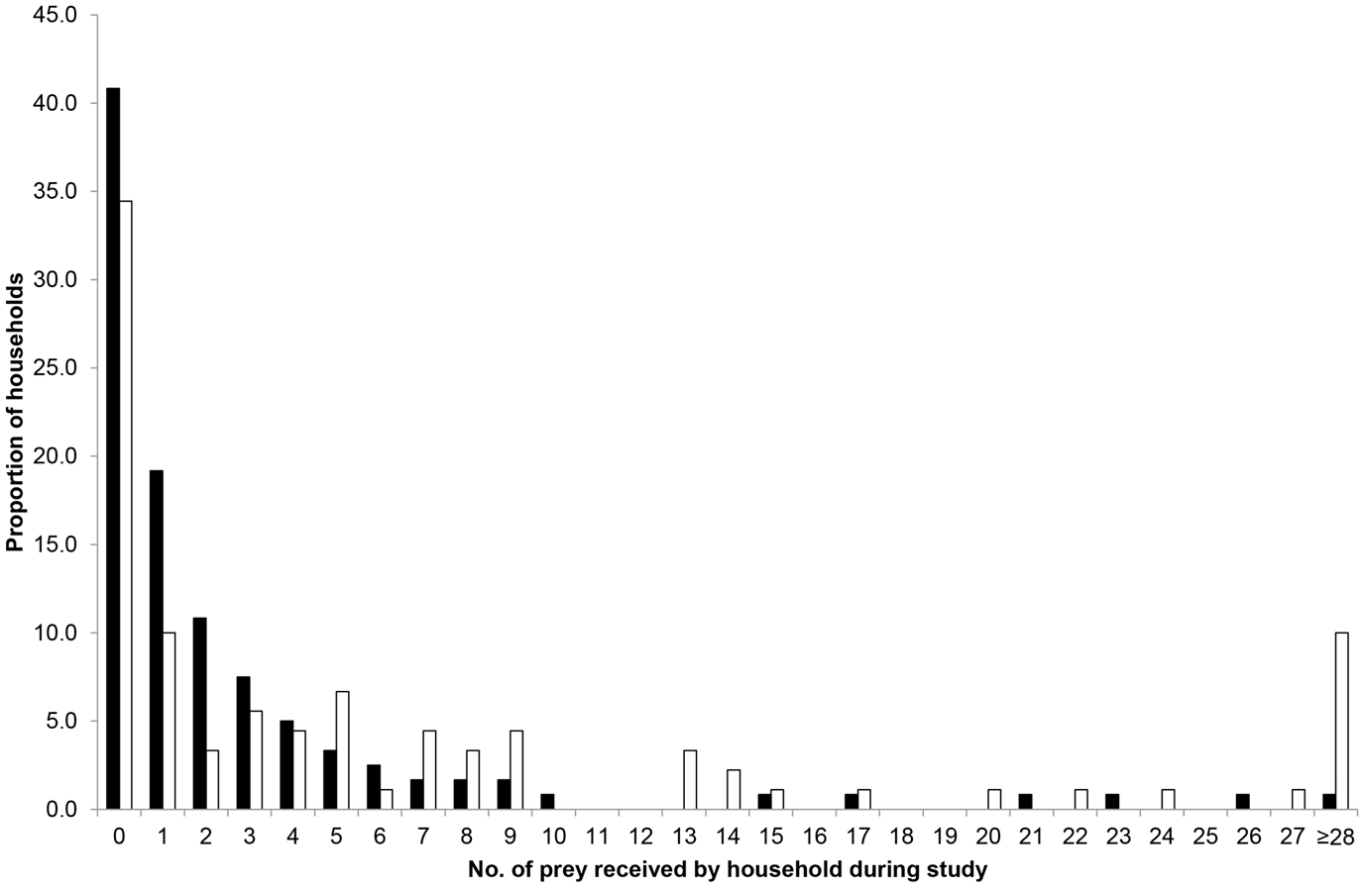
Frequency of number of prey returned in single-cat (black bars) and multiple-cat (white bars) households.

Despite the relative paucity of unequivocal evidence of a negative effect on prey populations, some authorities have suggested that “precautionary” management of urban cats should be implemented now while further studies are undertaken [33], [50]. Although pet cats are potentially more amenable to management than any other mammalian carnivore, it is important to note that there are marked inter-national differences in attitudes towards cats, the acceptability of different management strategies [27], [31], [33] and the likelihood of using legislative actions to effect change. For example, any approach within the UK would need to be adopted voluntarily as it is not conceivable that local or national government agencies would consider enacting any form of cat control given that cats do not need to be licensed and they are not perceived to be involved in any major zoonoses.

**Figure 2:**
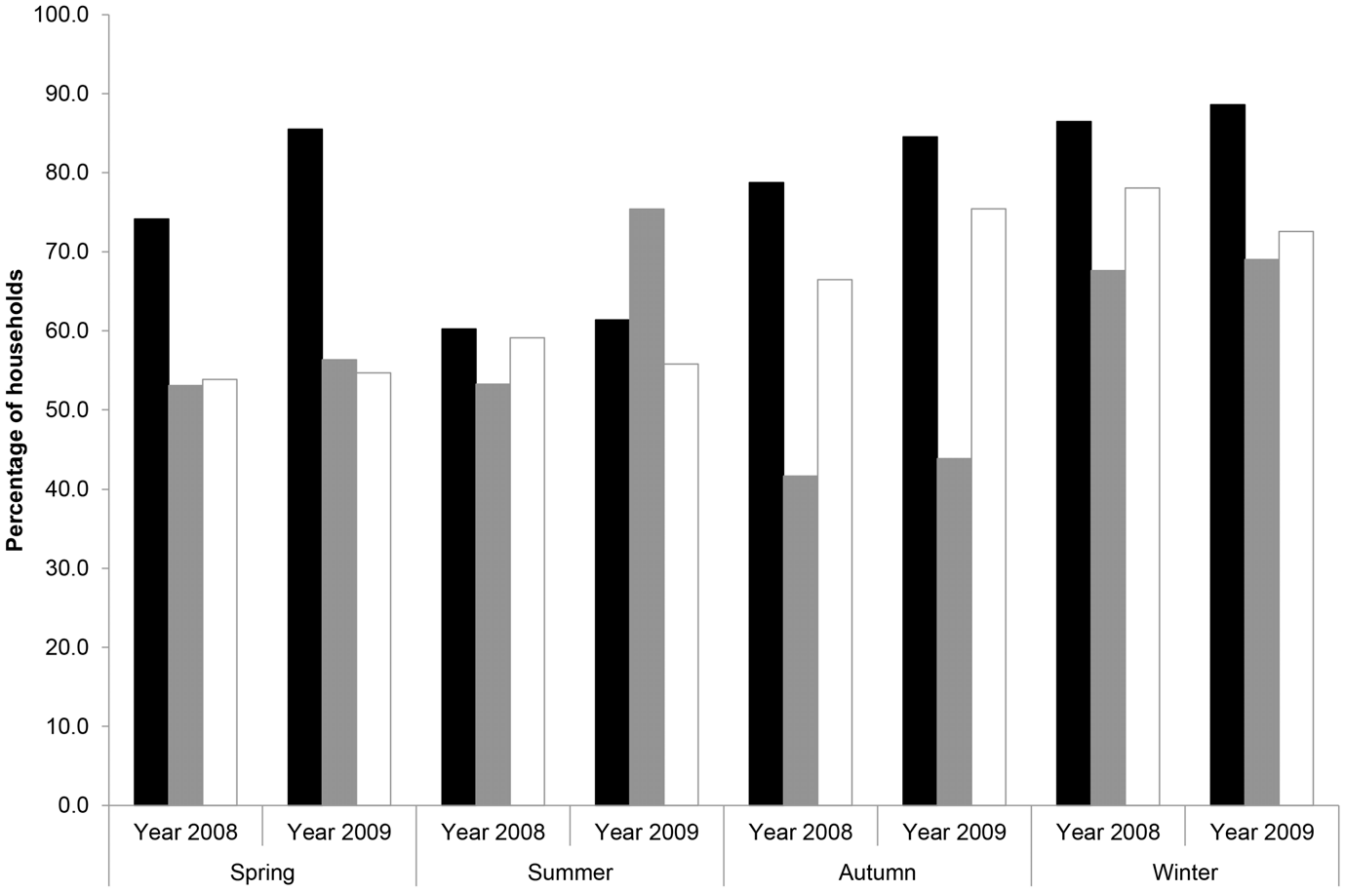
Percentage of single-cat (black) and multiple-cat (grey) households receiving zero prey each season, and the assumed minimum number of cats in multiple-cat households (white bars) delivering zero prey each season if prey were divided equally amongst individuals.

**Figure 3:**
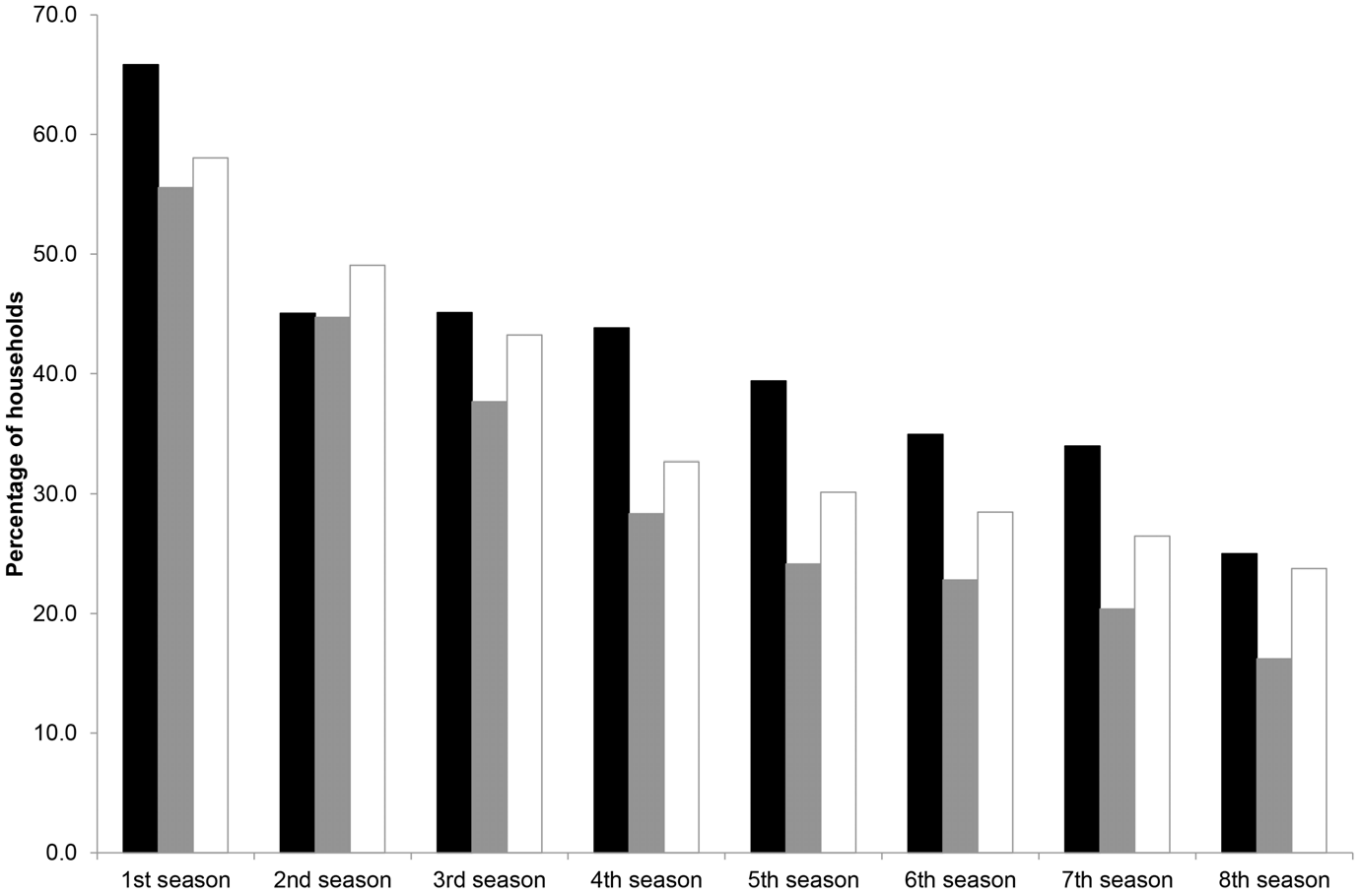
Percentage of single-cat (black) and multiple-cat (grey) households receiving zero prey each season, and the assumed minimum number of cats in multiple-cat households (open bars) delivering zero prey each season (see text for details) in relation to cumulative length of time in study.

Potential strategies for reducing cat density within the UK include an outright ban on owning cats, banning ownership in the vicinity of ecologically sensitive areas [34], [37], limitations on the number of cats an individual can keep and the imposition of a license or registration fee to increase the costs associated with ownership. All four measures are potentially controversial, as they may be seen as restricting personal liberty. Furthermore, license or registration fees could be seen as a form of tax on the poorest members of society, who could be “priced out” of receiving possible health benefits associated with pet ownership [51]. Strategies for manipulating cat behaviour to limit predation rates include restricting their ranging behaviour spatially and/or temporally through curfews or temporary confinement in the owner’s home, and/or the use of collar-mounted anti-predation devices [22], [23], [25], [26], [52]. Ultrasonic devices [53] and chemicals may also be used to deter cats. The most controversial method for reducing hunting efficiency is onychectomy (de-clawing) [28], [54], although this is banned in many countries except on medical grounds.

**Figure 4:**
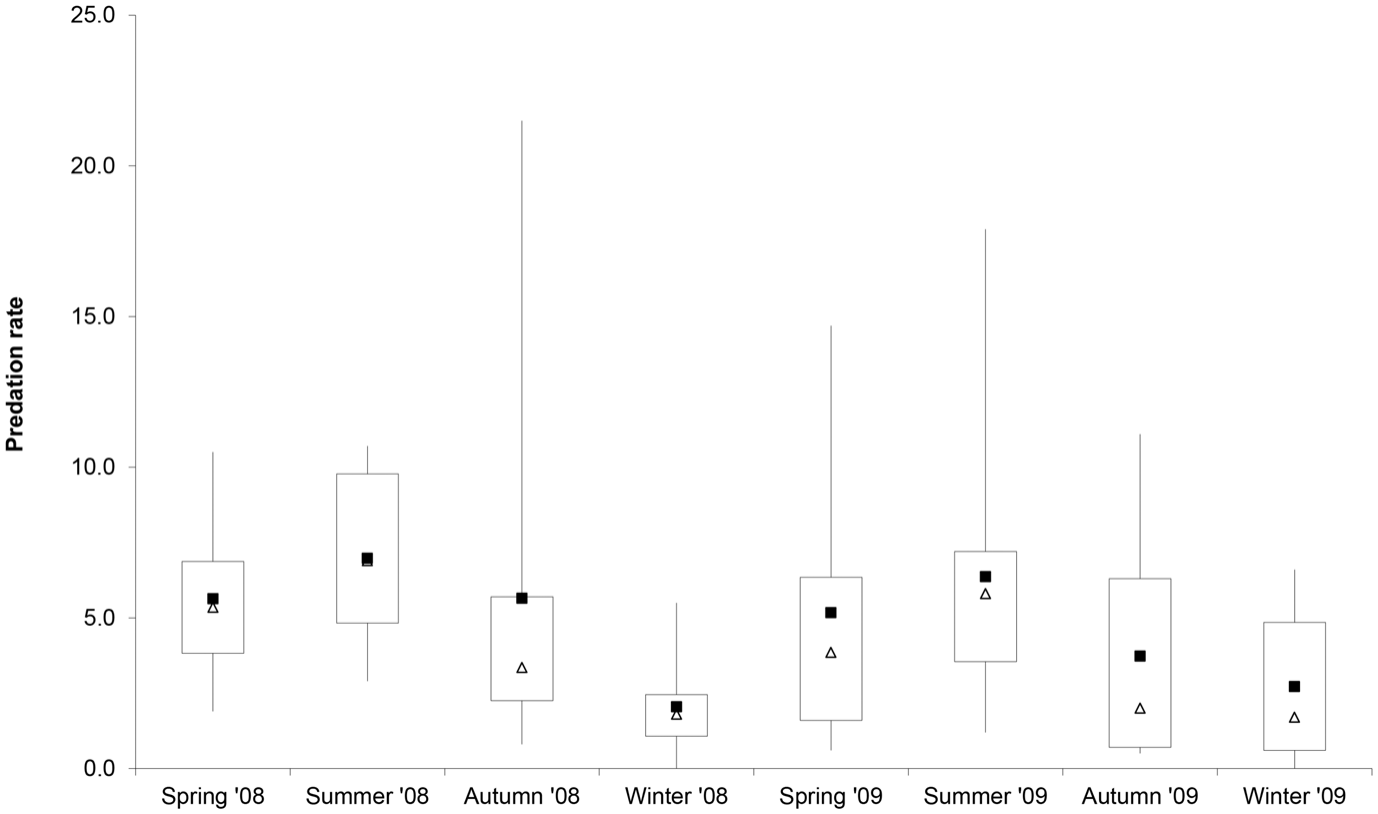
Boxplot of seasonal predation rates (prey cat ^−**1**^ **season** ^−**1**^ **).** Means and medians are represented by solid squares and open triangles respectively: boxes denote inter-quartile ranges; lines denote minimum and maximum values. Sample sizes are: spring 2008, N = 6 squares; summer 2008 to spring 2009, N = 9; summer 2009 to winter 2009, N = 12.

In summary, although there is a growing body of evidence that cats can affect prey populations in urban areas, this is currently limited to a relatively small number of studies that have been conducted primarily in the Southern Hemisphere where cats are a relatively new introduction. Furthermore, there are currently no data within the UK relating to the public acceptability of possible management strategies. Therefore, in this study we aimed to quantify cat density and individual predation rates within selected areas within the town of Reading, UK, to investigate (i) how the numbers of prey killed varies spatially within a single conurbation, (ii) whether per capita predation rates vary with cat density and (iii) to what degree predation rates vary intra-and inter-annually. Estimates of the numbers of prey killed were compared with the density and productivity of selected prey species to gauge (iv) whether predation by pet cats could be influencing the dynamics of prey populations in different locations. We then used a questionnaire survey to examine (v) current perceptions of the importance of predation by cats on urban birds, (vi) the acceptability of possible methods for managing pet cats in urban areas and (vii) how current cat-owning practices reflect these management practices and perceptions.

**Figure 5:**
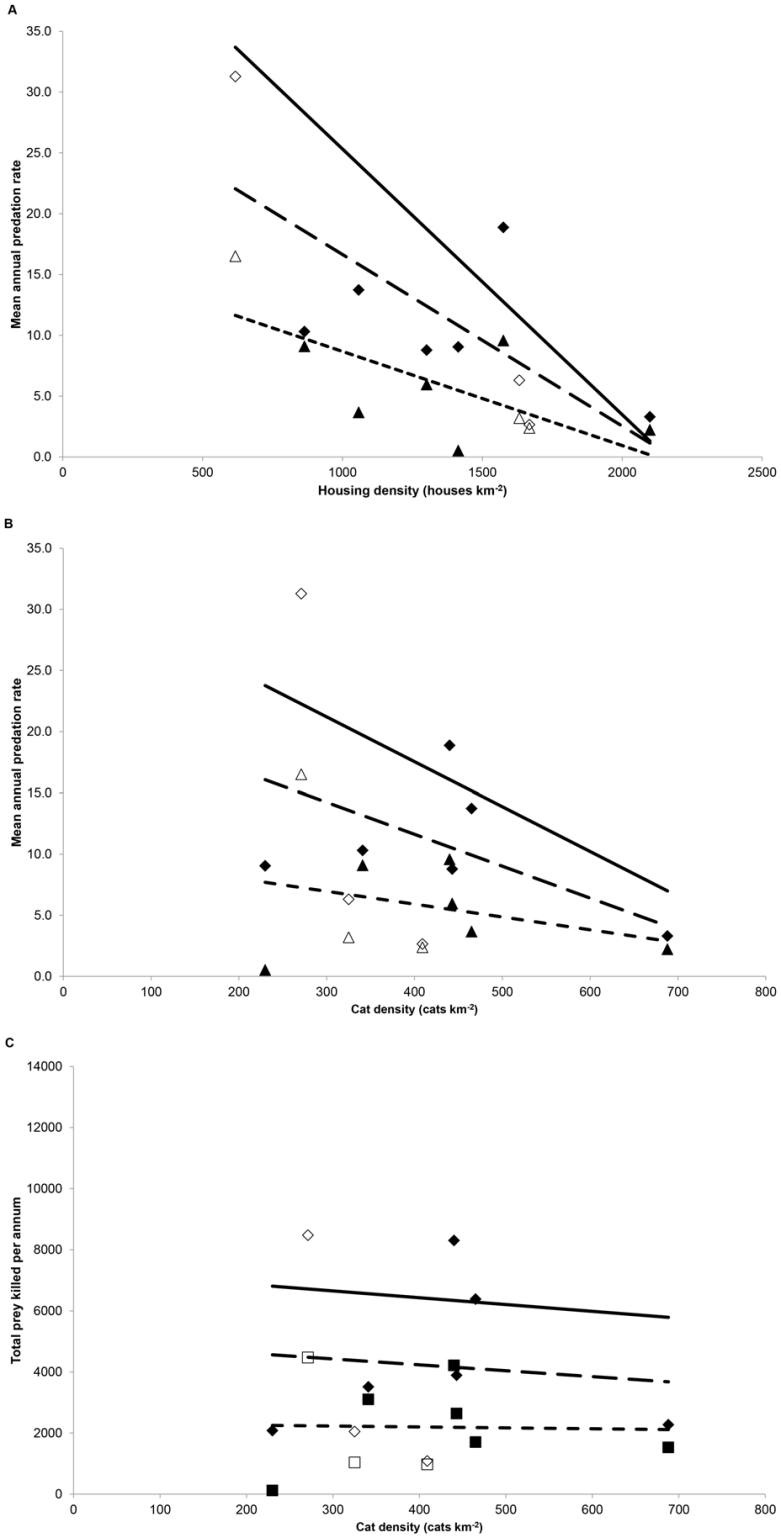
The relationship between (a) house and (b) cat density and annual predation rates and (c) cat density versus total number of prey killed annually. Diamond symbols & large dashed line denote relationship with mammals: triangles & small dashed line denote relationship with birds. Solid line denotes relationship with both groups combined; for clarity, symbols for both groups combined have been omitted. Solid symbols denote means for survey squares studied over two years; open symbols denote squares studied in one year only.

## Materials and Methods

The study was conducted in Reading, UK (51°27′N, 0°58′W) during 2008–2010. The town covers *c*. 55 km^2^ and has a population of 230,000 people. Data on cat density, predation rates and attitudes to management strategies were collected from randomly selected Ordnance Survey 1-km squares covering a range of housing densities and socio-economic classes.

**Figure 6:**
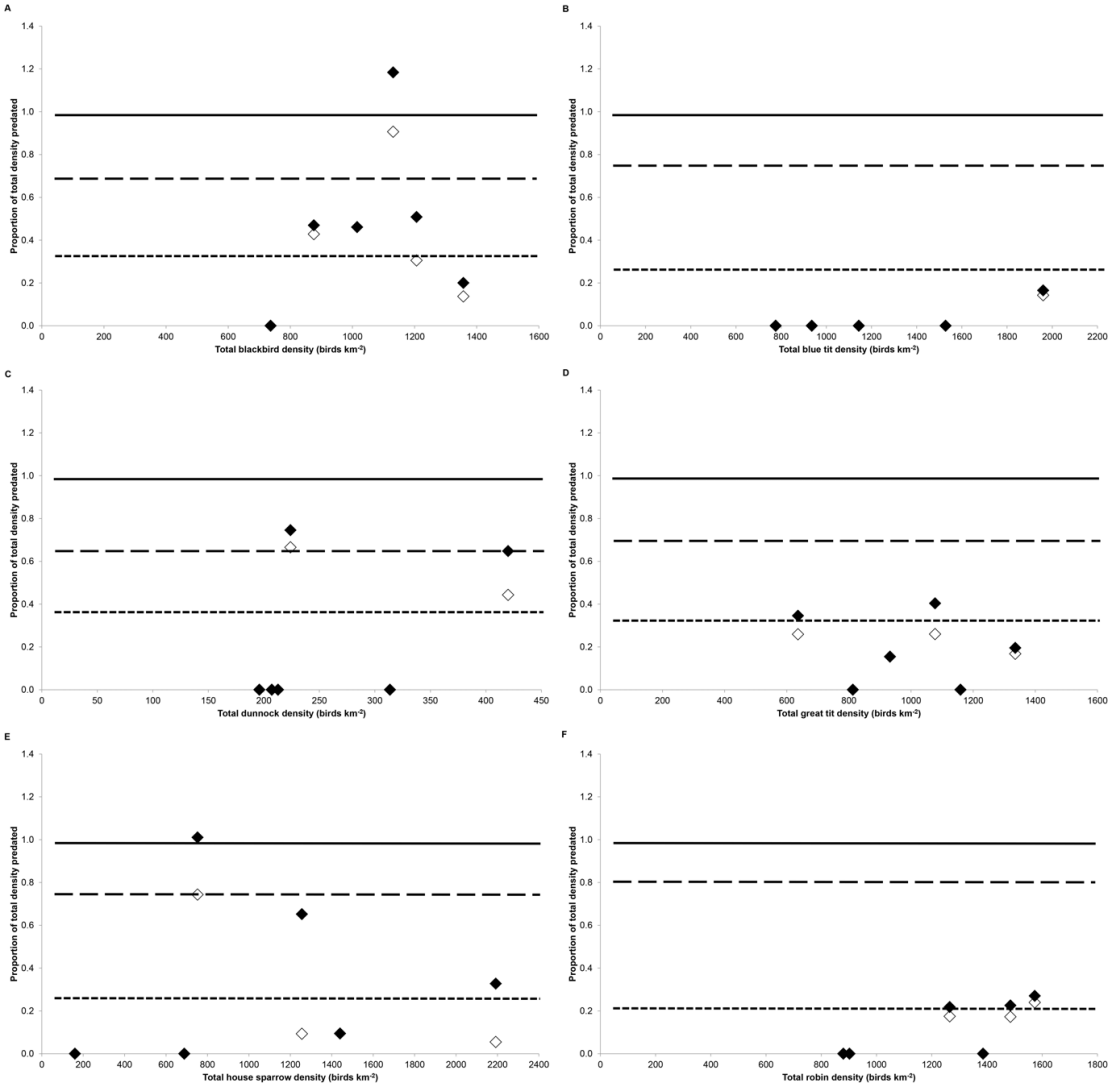
Relationship between the density of (a) blackbirds, (b) blue tits, (c) dunnocks, (d) great tits, (e) house sparrows and (f) robins and the estimated proportion of the population killed (N = 6 squares). The solid line denotes the total population (proportion = 1): the lower and upper dashed lines indicate the proportion of adults and juveniles in the population respectively. Open and closed symbols denote the estimated minimum and maximum number killed expressed as a proportion of the population.

### Predation Study

To maximize the number of volunteers recruited into the predation study, leaflets were hand-delivered to every house in nine survey squares requesting information on: the number of cats owned; whether they had been neutered, wore a bell or other anti-predation device; whether they were allowed out during the day only, night only, or both; and whether they would record prey brought home by their pet(s). Householders were asked to leave the completed form on their doorstep for collection (“original” phase). As this approach may provide a biased sample of cat-owners, the proportion of cat-owning households in non-responding houses was estimated by contacting 50–100 randomly selected addresses in each square (“follow-up” phase), this number having been shown to be sufficient to estimate abundance across a broad range of densities [11]. Density in each square was estimated as the product of the number of unsurveyed households, the proportion of households containing cats in the follow-up phase and the mean number of cats per cat-owning household identified in both phases plus the number of individual cats identified in the original and follow-up phases. Although the number of cats per household is typically not normally distributed, mean values have tended to be used in similar studies since medians would intrinsically underestimate cat density: we present estimates of cat density derived using both mean and median values.

**Figure 7:**
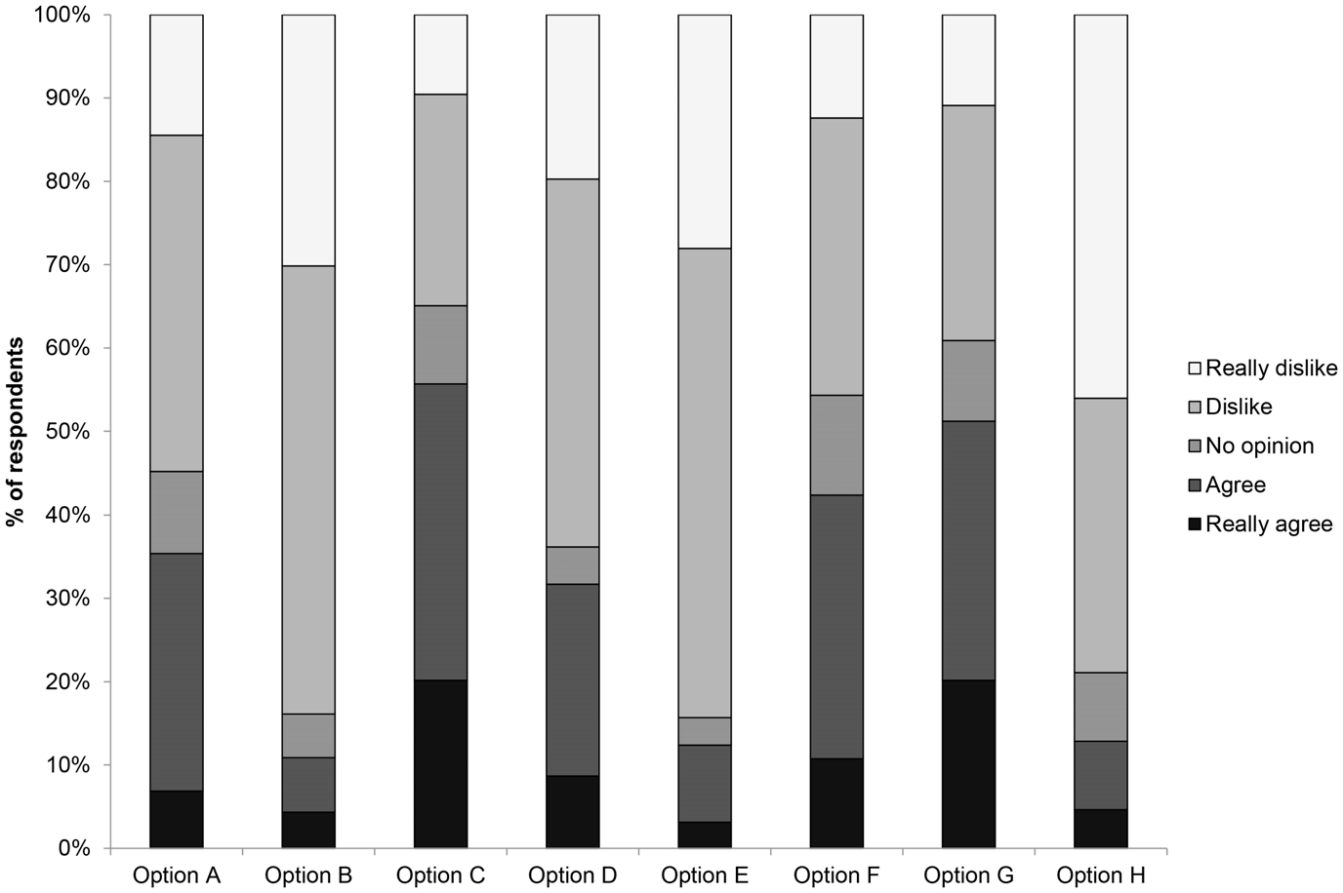
Percentage of respondents that agreed with, disagreed with or had no opinion of potential cat management options: A - people living near ecologically sensitive areas should not own cats; B – town/city residents should not own cats; C – cats should be fitted with a bell or other anti-predation device; D – cats should remain within their owner’s garden; E – cats should be confined during the day; F – cats should be registered with the local council; G – cats should undergo compulsory sterilisation; H – cats should be de-clawed.

**Table 2:** Summary of results of binary logistic regression models examining the acceptability of eight different cat management options (see Figure 7) (N = 665 interviewees).

Variable	Term	Cat management options							
		Option A	Option B	Option C	Option D	Option E	Option F	Option G	Option H
SITE(Site 1)	P	NS	NS	NS	NS	0.001	0.022	NS	NS
Site 2	OR	–	–	–	–	–	–	–	–
Site 3	OR	–	–	–	–	–	–	–	–
Site 4	OR	–	–	–	–	–	2.21	–	–
Site 5	OR	–	–	–	–	–	–	–	–
Site 6	OR	–	–	–	–	4.21	–	–	–
Site 7	OR	–	–	–	–	–	–	–	–
Site 8	OR	–	–	–	–	–	–	–	–
Site 9	OR	–	–	–	–	2.84	–	–	–
Site 10	OR	–	–	–	–	–	–	–	–
SEX(Male)	P	NS	NS	NS	NS	NS	NS	0.002	NS
Female	OR	–	–	–	–	–	–	1.65	–
AGE(<40 years)	P	0.009	NS	0.039	NS	NS	0.007	NS	<0.001
≥40 years	OR	0.64	–	0.68	–	–	0.60	–	0.47
CAT(Don’t own)	P	0.007	<0.001	<0.001	<0.001	<0.001	NS	NS	<0.001
Do own cat	OR	0.64	0.33	0.43	0.28	0.21	–	–	0.26

NS denotes term was removed in backward stepwise elimination procedure. OR denotes odds ratios: for variable SITE, only the odds ratios for sites differing significantly from the reference category (SITE 1) are presented.

In addition to these nine survey squares, the methodology used to survey residents about their attitudes to management options (see below) permitted cat density to be estimated in seven further squares. In these, ≥50 randomly selected householders were interviewed face-to-face. Cat density was estimated as the product of the total number of houses, the proportion of households containing cats and the mean number of cats per cat-owning household. Since the proportion of households owning cats and the number of cats per cat-owning household may vary with socioeconomic status, correlation analysis was used to investigate whether housing density was associated with the proportion of cat-owning households, the mean number of cats per cat-owning household and total cat density across the 16 survey squares.

### Return Rates

Predation patterns were estimated in 12 squares during 2008–2009. Individual squares were studied for 8 (N = 6), 7 (N = 6) or 3 (N = 3) seasons. Seasons were defined as: spring (March-May); summer (June-August); autumn (September-November); winter (December-February). Data were recorded for six weeks each season to maximize participation rates. Mean seasonal return rates were calculated in each square as the sum of dead and live prey returned divided by the number of cats under study. Live prey have been incorporated into estimates of mortality as most are likely to have perished. For example, one study reported that *c*. 40% of cat-caught birds entering Dutch wildlife hospitals died from their injuries with the remainder subsequently perishing from infections [55].

Prey returns were used to examine the frequency with which householders received no prey each season (“zero return”) because owners often cite the fact that their cats do not return prey as evidence against an effect on wildlife. Three measures were considered within each season and in relation to the cumulative length of time under study: the proportion of (i) single-cat households and (ii) multiple-cat households receiving no prey; and (iii) the minimum number of cats in multiple-cat households delivering no prey. The latter was derived by assuming that individuals within multiple-cat households contributed equally to returning prey i.e. if three prey items were returned to a household containing five cats, we assumed that two cats did not return any prey that season.

### Predation Rates

Predation rates were calculated by multiplying return rates by 2.17 (because householders recorded returns for only six weeks each 13-week season) and a conversion factor relating to the proportion of prey killed that were returned home. Estimates for the latter vary from 50% [44], to 30% [18] and 12.5% [45]. However, in [44] this was derived as the proportion returned both to the author’s home and to a “delivery area” observed through a window: as our study relied on prey brought into the owner’s home, applying this figure would tend to under-estimate predation rates. Therefore, we have elected to use a return rate of 30% (i.e. a conversion factor of 3.3), as this would generate minimum estimates of predation rates.

As not all cats were studied in all seasons due to differences in the timing of recruitment, pet mortality and volunteer drop-out, predation rates were calculated seasonally to maximize sample sizes. Mean predation rates within each square each season were multiplied by cat density to estimate the total number killed, and summed across seasons to estimate the number killed annually. Seasonal differences in predation rates were quantified using data from those cats from single-cat households studied for ≥4 seasons; where an individual had been studied in the same season for two years, predation rates for that season were averaged. Data were analysed using a repeated-measures ANOVA, with the Greenhouse-Geisser correction for lack of sphericity.

Although participants were asked to keep all dead animals for identification, two groups of unidentified prey were commonly recorded that needed to be accounted for when estimating predation rates on individual species: unidentified small mammals (taken to be mice, voles and shrews) and unidentified birds (taken to be passerines as householders did record other groups as e.g. pigeons, ducks). Estimates of minimum numbers killed in each square used only those returns which had been identified definitively i.e. assuming that unidentified prey were species other than those in the list of identified prey. However, previous studies where owners were asked to identify dead prey have indicated that householders are often not able to distinguish even common species [12], [13], [20]. Estimates of maximum numbers killed were therefore derived assuming that unidentified prey were species in the list of identified prey. Unidentified prey were redistributed in direct proportion to the distribution of identified prey i.e. if species×represented 50% of identified small mammals, then 50% of unidentified small mammals were assumed to be species X.

### Potential Effect of Cat Predation

The breeding density of six species was estimated in six squares using distance sampling [56]: blackbird (*Turdus merula*), blue tit (*Cyanistes caeruleus*), dunnock (*Prunella modularis*), great tit (*Parus major*), house sparrow (*Passer domesticus*) and robin (*Erithacus rubecula*). Point counts were conducted during May–July 2008 from 16 pseudo-random locations within each square; locations were >200 m apart to minimize the chance of double-counting. One square was surveyed each day: locations within the square were visited in a random order. Each square was visited three times. Surveys commenced 30 min after sunrise. Birds were recorded within five distance bands (0–10 m, 10–20 m, 20–30 m, 30–40 m, 40–50 m) from the observer for 5 min, after allowing for a 5 minute settling down period. Birds were identified visually and acoustically. Data were analysed using Distance 5 Release 2 [56]; global detection functions were calculated for each species across the six squares. Akaike’s Information Criterion was used to select the best of several candidate models fitted to each species’ data. Estimated density was divided by two to derive the number of breeding pairs, as there is no evidence of sex bias in the likelihood of detection within the species surveyed [57]. Annual juvenile production per breeding pair was estimated from published data: blackbird, 3.8; blue tit, 6.0; dunnock, 3.6; great tit, 4.3; house sparrow, 6.0; robin, 9.0 [12].

Spearman’s rank order correlations were used to compare the relationship between total bird density and the estimated maximum number of each species killed. Although there was slight variation in the relative ranking of estimated minimum and maximum numbers (due to the number of unidentified passerines returned in each square and how these were partitioned to estimate maximum numbers killed), these did not affect the pattern of results. Consequently, for brevity, we have reported only those results for maximum estimates.

### Cat Management Study

Attitudes to management options were quantified using face-to-face interviews of ≥50 randomly selected householders within ten 1-km squares. Householders were asked to indicate the acceptability of eight management options on a five-point Likert scale in response to the introductory statement: “If it was shown that cats were having a severe impact on garden birds and some form of management solution was considered necessary, please indicate whether you strongly agree, agree, disagree, strongly disagree or have no opinion about each of the following”: (a) people living in ecologically sensitive areas should not be allowed to own a cat; (b) no one living in a town or city should be allowed to own a cat; (c) all cats should be fitted with a bell or other anti-predation device; (d) cats should not be allowed to roam outside their owner’s garden; (e) cats should not be allowed out during the day; (f) cats should be registered with the local council; (g) cats should undergo compulsory sterilisation; (h) cats should be de-clawed.” Statements (f) and (g) were included partly to investigate attitudes towards aspects of pet ownership/management that have previously been implemented within the UK, or that have been the subject of campaigns by animal welfare organisations. We focussed on the possible effect on birds as this is the taxonomic group for which cat management is most widely discussed by the national media in the UK.

Because of relatively low samples for some combinations of variables, respondents were grouped into two categories for statistical analysis: those that strongly agreed, agreed or had no opinion were combined (“acceptable”) and compared with those replying that they disagreed or strongly disagreed (“not acceptable”). The effect of survey square (SITE), gender (SEX), age (<40 years, ≥40 years) and whether the interviewee owned a cat or not (CAT) on the acceptability of each management option was quantified using binary logistic regression. These two age groups were selected to represent people that would have had some experience or memory of licensing pets (dog licenses were abolished in the UK in 1987) versus those that did not. Initial models included all two-way interaction terms where possible; models were simplified using a backwards stepwise elimination procedure (α = 0.05).

Perceived effects of cats on wildlife were assessed using two data sets. First, those people interviewed in the management survey were asked whether they considered cats to be a nuisance and why: interviewees were not given prompts and were allowed to list multiple reasons; differences between these divisions were compared using chi-squared tests. Second, a postal survey of the cat owners participating in the predation study were asked whether they thought that cats have no, small, moderate, large or very large negative influence on local bird populations.

Patterns of sterilisation, wearing of collar-mounted anti-predation devices and temporal ranging behaviour (day only, night only, day and night) were quantified from original and follow-up surveys used to estimate cat density. Reasons for not wearing bells were examined in the postal survey of cat owners: respondents were asked whether their cats wore a bell and, if not, to indicate why not; multiple responses were permitted. As the original and follow-up surveys had been used to recruit cat owners for the predation study, there is some overlap between these two samples.

All statistical analyses were conducted in Minitab (v.15; Minitab, Inc., State College, PA). Data were checked to ensure that they conformed to the assumptions of each test. As not all questions were asked during each questionnaire survey and because not all interviewees answered all questions, sample sizes vary accordingly. This project has been subject to ethical review according to the procedures specified by the School of Biological Sciences Ethics and Research Committee, University of Reading and has been given a favourable ethical opinion for conduct.

## Results

There was a significant difference between original and follow-up houses in the proportion of householders owning cats (paired *t* test: *t*_8_ = 6.17, P<0.001; N = 9 squares: Table 1) but there was no significant difference in the mean number of cats per cat-owning household (paired *t* test: *t*_8_ = 0.92, P = 0.385) or the number of households owning one, two or ≥three cats (chi-squared test: χ^2^_30_ = 27.55, N = 16 squares, P = 0.594). Overall, 23±7% of householders within each square owned cats (range: 10–34%), with a mean of 1.54 cats per cat-owning household (range: 1.30–1.91).

Estimates of cat density based on mean values (463±208 cats km^−2^) were markedly greater than those based on medians (324±130 cats km^−2^: Table 1). House density was not correlated with the proportion of households owning cats (r = 0.039, N = 16, P = 0.887) or the mean number of cats per household (r = −0.263, N = 16, P = 0.325), but was significantly positively correlated with total cat density (r = 0.727, N = 16, P = 0.001). Cat density, therefore, increased predominantly as a function of housing density.

### Return Rates

Data were collected from 348 cats from 211 households in 12 squares. On average, households were studied for 5.4 seasons (range: 3–8) with a mean of 24.3 cats (range: 11–45) studied per square each season. Eleven mammal, 21 bird, one amphibian and two reptile species were recorded (N = 988 dead and N = 162 living prey: see Figure S1). Mammals accounted for 65% of prey returned, with the wood mouse (*Apodemus sylvaticus*) contributing 40% of all records; birds contributed 30% of records.

Individual return rates were highly variable (Figure 1). In single-cat households, 41% of householders received no prey and only 22% returned ≥4 prey (range: 1–28). Comparable figures for multiple-cat households were 34 and 47% (range: 1–68), respectively. There was a significant positive correlation between the total number of prey received each season by households studied in both years (spring: N = 77 households, r = 0.499, P<0.001; summer: N = 115, r = 0.670, P<0.001; autumn: N = 116, r = 0.573, P<0.001; winter: N = 112, r = 0.628, P<0.001).

Zero returns to single-cat households were lowest in summer (61% of households) and highest in winter (88%), with values being slightly higher in 2009 (Figure 2); on average, 78% of householders received no prey each season. The pattern for multiple-cat households was markedly different, being lowest in autumn and with substantial inter-annual variation in the summer season: the pattern in these houses was much more similar to that observed in single-cat households if it was assumed that prey were delivered evenly by individual cats (Figure 2). Combining data from both household types, the approximate minimum number of cats not returning prey each season was: spring, 58%; summer, 58%, autumn, 73%; and winter, 76%. But there was a substantial decline in the likelihood that cats did not deliver any prey with increasing length of time studied: 66% of cats in single-cat households did not return any prey in their first season; this declined to 44% and 25% after four and eight seasons, respectively (Figure 3). Comparable figures for cats in multi-cat households were 58, 33 and 24%, respectively. This would suggest that the majority of cats did kill prey to some degree.

### Predation Rates

Mean seasonal predation rates based on all cats studied ranged from 1.9–6.2 prey cat^−1^, being highest in summer and lowest in winter (Figure 4). This pattern was also evident for those cats (N = 73) studied for ≥4 seasons (repeated-measures ANOVA: *F*_1.93, 139.15_ = 8.88, P<0.01; *post hoc* groups: summer >spring = autumn = winter). However, there was marked differences in seasonal predation rates both amongst squares and/or between years. For those nine squares surveyed for four seasons in 2008 and/or 2009, the mean annual predation rate was 18.3 prey cat^−1^ year^−1^ (range: 5.6–47.7).

There was a significant negative correlation between housing density and annual predation rates on birds (r = −0.699, P = 0.036: N = 3 squares studied in one year only and 6 squares averaged over two years of study) and mammals (r = −0.719, P = 0.029), and mammals and birds combined (r = −0.732, P = 0.026: Figure 5a). Similarly, there was a negative relationship with cat density (Figure 5b) but these were not significant (mammals: r = −0.394, P = 0.294; birds: r = −0.281, P = 0.465; total: r = −0.363, P = 0.336). As a consequence, there was a marginal decline in the total numbers of mammals and birds killed with increasing cat density (Figure 5c). There was, however, no significant correlation in predation rates between years for mammals (r = 0.501, P = 0.311), birds (r = 0.389, P = 0.446) or both groups combined (r = 0.559, P = 0.249) for those six squares studied in 2008 and 2009.

There was no significant correlation between total cat density and the estimated maximum number of prey killed for blackbirds (r_S_ = 0.314, P = 0.544), blue tits (r_S_ = 0.664, P = 0.150), dunnocks (r_S_ = 0.541, P = 0.268), great tits (r_S_ = −0.058, P = 0.913) or house sparrows (r_S_ = 0.464, P = 0.354), in part because predation on these species was often not recorded in the squares surveyed, but there was a significant correlation for robins (r_S_ = 0.820, P = 0.046). The maximum numbers killed exceeded a number equivalent to the proportion of adults for blackbirds in four survey squares, for house sparrows and robins in three squares, and for dunnocks and great tits in two squares; rates for blue tits did not exceed this value in any survey square (Figure 6).

### Cat Management

Including individuals that expressed no opinion, the acceptability of management techniques ranged from 15.7% (daytime curfew) to 65.1% (wearing of anti-predation devices) of interviewees (Figure 7). Registration with the council (54.3%) and compulsory sterilisation (60.9%) were the only other options acceptable to the majority of people surveyed (Figure 7). However, cat-owners were significantly less likely to accept most management options (Table 2).

Cats were considered to be a nuisance by 19% of cat-owners (N = 248) and 46% of non-owners (N = 420). The most commonly given reason was defaecating in their garden, but this was less frequently cited by owners (44% versus 68%: χ^2^_1_ = 15.03, P<0.001). Digging (17% versus 33%: χ^2^_1_ = 4.30, P = 0.038) and urinating (22% versus 29%: χ^2^_1_ = 1.06, P = 0.303) were also common complaints. There was no significant difference between owners and non-owners in terms of a perceived effect on wildlife (37 versus 27%: χ^2^_1_ = 1.91, P = 0.168), these figures equating to 7 and 12% of all owners and non-owners, respectively. In comparison, 16% of cat-owners in the postal survey (N = 110) stated that they thought cats had no effect on local bird populations whereas 51, 25, 5 and 2% thought they had a small, moderate, large or very large effect, respectively.

Ninety-six per cent of cats were neutered (N = 728 cats, 457 households): 2, 27 and 71% were allowed out during the day only, night only or day and night, respectively; 24% wore a bell or sonic device. In the postal survey (N = 205 cats, 113 households), 71% did not wear a collar, 9% wore a collar with no bell and 20% wore a collar with a bell. Seventy-four owners indicated why their cats did not wear a collar: 28% expressed concerns over the cat’s safety; 28% said that they had failed to replace a shed collar; and 26% had removed the collar because of injury or signs of distress.

## Discussion

Cat densities and the general patterns of predation recorded in this study were broadly comparable to those observed in other studies: (i) cat density increased with housing density [11]; (ii) marked variation in the numbers of prey returned by individual cats (range 0–28, with only 22% of cats returning ≥4 prey annually); (iii) most householders did not receive any prey each season, although the proportion of householders receiving zero prey declined from 66% after one season to 25% after eight seasons indicating that the majority of cats did return prey at some point in their lifetime; (iv) small mammals were the commonest prey group, accounting for 65% and 49% of dead and live prey respectively; and (v) the highest predation rates were observed in spring and summer when prey were breeding. However, even assuming that prey returned alive would have perished, the mean predation rate (18 prey cat^−1^ year^−1^) is among the lowest recorded to date (14–302 prey cat^−1^ year^−1^: [12]–[26]).

In addition, annual per capita predation rates declined with increasing cat density, ultimately resulting in a reduction in the total number of birds and especially mammals killed annually. In contrast, avian density within the UK has been shown to increase generally within the range 620–3201 houses km^−2^ [48], which was the range present in the current study (Figure 5). This would suggest that the proportion of total avian prey killed by cats would decline marginally as housing density increases beyond ∼1,500 house km^−2^. However, the pattern of change of individual species within this range is variable, with many reducing in density [48]. Consequently, the effect on individual species will very much depend upon their pattern of changing abundance and concomitant changes in the functional response of pet cats.

The estimated numbers of the six focal bird species killed were greater than the proportion of adults in the corresponding prey population on 14 of 36 occasions. Gauging the influence of this level of mortality on prey populations is problematic. For example, simulation modelling approaches [17], [58] have relied upon creating population growth models onto which observed levels of cat predation are imposed to determine whether populations persist or decline. However, due to a paucity of data on the demographics of urban bird populations, such studies have had to use data from a wide range of countries and populations that could have already been subject to cat predation. Despite these limitations, the “half-predation level” model developed for blackbirds in Dunedin, New Zealand [17] is based on a mortality rate ((10,255/15,497)*0.5 = 0.33) broadly comparable to those observed for blackbirds in several of our sites, and which indicated that such predation levels are likely to result in long-term declines. Similarly, although there are certainly differences between urban and rural populations with respect to density and productivity [59], some of the mortality rates observed for were also broadly comparable with annual mortality rates derived from models for declining populations of the same species in farmland habitats (e.g. blackbird: 0.37–0.46; dunnock: 0.53–0.65; house sparrow: 0.50–0.67; [60]).

Furthermore, our estimates are based on some assumptions that could have under-estimated predation rates. For example, the bird surveys were conducted at a time where it is plausible that some individuals may have already bred: dividing bird density by two to derive the number of breeding pairs would, therefore, have overestimated productivity; consequently the estimated number of birds killed would have been a greater proportion of the prey population. Similarly, assuming that cats returned 30% of the prey killed [18] may be a significant over-estimate [45], [61], again suggesting that actual predation rates were substantially higher. These results are, therefore, consistent with the notion that the mortality caused by free-ranging pet cats can be substantial for some prey species in some circumstances [12]–[15], [17], [58]. The marked spatial variation observed would also suggest that using data at the spatial scale utilised in this study to extrapolate city-wide predation rates are likely to be equivocal without much greater information about the relationships between cat density, prey density and predator functional responses.

In addition to spatial variation, there was also marked temporal variation in the numbers of prey killed. For example, the relative distribution of seasonal predation rates within the same survey square was not consistent between years and these frequently differed two-fold between years, although this variation was not consistent with chronological year. As a result, there was no significant correlation between annual predation rates for those squares surveyed for two years. Such variation could have arisen for a number of reasons, but one possible explanation is that because pet cats are not reliant on wild prey for their survival, predation is predominantly stochastic and particularly influenced by factors at a localised scale. These could include fine-scale changes in the distribution of both prey (e.g. in response to artificial feeding stations and nesting sites within the ranges of individual cats) and cats (e.g. as a consequence of turn-over in house ownership, purchase of new pets) and variation in factors that affect the propensity (e.g. age, neuter status: [15], [21]) and opportunity (e.g. localised weather patterns) of cats to hunt. Given that the numbers of prey killed by individual cats are typically low, even small-scale changes in the numbers killed would have a substantial influence on estimated predation rates. As a consequence, predation rates, and the method employed to estimate them, would be very sensitive to a range of processes operating on individual animals at very fine scales within a very heterogeneous landscape. Furthermore, the implicit assumption of studies of this sort is that prey returned home accurately reflects patterns of predation in the wider environment. This certainly requires further investigation given the marked variation in reported values for the proportion of prey returned home [18], [44], [45] and that cats undoubtedly preferentially consume some species and return others [61]. These facets of cat behavior could be quantified using animal-mounted video systems [62].

These caveats aside, this study and others [12], [13], [45] suggest that consideration of methods for managing urban cat populations within the UK would be appropriate at this time. Given that any management action(s) introduced in the UK would have to be adopted voluntarily, their uptake would be dependent on whether urban residents perceive cat predation as a factor warranting management and the acceptability of different actions to owners in particular but also the wider public as a whole.

Public perceptions of the effect of cats were somewhat contradictory. Few cat-owners (7%) and non-owners (12%) listed consequences for wildlife as a nuisance value in response to open-ended questions in face-to-face surveys, but 32% of owners in the predation study considered cats had a moderate or greater effect on wildlife when asked to specifically consider this question. These differences are potentially associated with the different question forms, but would appear to indicate that most householders do not believe predation is currently a significant problem and also that cat-owners may be willing to overlook the “fact” that their pets may be affecting local wildlife. One possible reason for the latter is that most owners only get to see a small part of a much bigger picture i.e. they receive little or no prey from their own pets, and do not appreciate the possible combined effect of the pet population as a whole, although in one study owners tended to over-estimate their pets’ predation rates [49]. Furthermore, these percentages are substantially lower than comparable figures on the proportion of respondents that considered urban cat predation to be a problem in both Western Australia (63–95% [33]; 77% [31]) and Texas (73–77% [27]), indicating that the perception of the role of cats as predators is markedly different at an international level.

The most popular option for potentially managing cat predation was fitting pets with a collar-mounted anti-predation device. These have been shown to be effective in lowering predation rates [22], [23], [25], [26], [52], [63], although the reduction may not be of sufficient magnitude to prevent prey declines in all circumstances [17]. Furthermore, there was a significant difference in acceptability between cat-owners (52%) and non-owners (73%) which was reflected in current patterns of collaring; <25% of cats were fitted with a collar and bell. In part, this reflected perceived risks from collars. For example, 28% of owners of uncollared cats were concerned about it getting caught and 26% had removed one because their pet had been distressed or injured. Achieving high levels of collaring would pose some logistical problems, but the targeting of those individual cats that kill large numbers of prey might be an effective measure.

Few interviewees considered an outright ban on cat ownership (16%), a daytime curfew (16%) or confining animals to their owner’s garden (36%) acceptable, but a greater proportion stated that a ban on ownership near ecologically sensitive areas was reasonable (45%). Despite this aversion to curfews, they do potentially represent an effective way to manage cat activity and some owners already restrict their pet’s ranging behaviour, e.g. 28% are confined at night but most (70%) are allowed to roam freely day and night. From a conservation perspective, curfews would most likely be effective at dawn and dusk (i.e. during the day) as this is when birds are most likely to be taken but would probably have little influence on predation of small mammals. Given the low level of acceptability, however, daytime curfews are unlikely to be adopted voluntarily within the UK without additional publicity of their potential benefits.

In summary, our data suggest that the numbers of birds killed by pet cats in some localities within urban areas may be sufficiently large that they could be negatively affecting prey populations. In comparison, most urban residents did not consider cat predation to be a significant problem. Of the management actions explored, only fitting with collars was identified as acceptable to the majority of the general public, but it was less acceptable to cat-owners probably because of the perceived risk of getting caught and the distress/injury caused by collars. Curfews, which are possibly the easiest way to manage cat predation, were acceptable to only a small minority of people. However, there are a number of methodological issues which require further investigation and validation, most notably determining the proportion of different prey species returned home and its sensitivity to fine-scale factors affecting individual cats.

## Supporting Information

Table S1 **Latin names of species listed in the manuscript and a summary of the number of animals returned dead and alive during the study.** The majority of animals returned dead were retained for positive identification; those released alive were identified by householders, so identifications may not always have been accurate.
(DOCX)
