# Supplementary material for: Spatio-Temporal Variation in Predation by Urban Domestic Cats (Felis catus) and the Acceptability of Possible Management Actions in the UK
Source: PLoS One. 2012 Nov 16;7(11):e49369. doi: 10.1371/journal.pone.0049369 (PMC3500283; doi:10.1371/journal.pone.0049369)
Supplement: Table S1 — Latin names of species listed in the manuscript and a summary of the number of animals returned dead and alive during the study. The majority of animals returned dead were retained for positive identification; those released alive were identified by householders, so identifications may not always have been accurate. (DOCX) [file pone.0049369.s001.docx]

**Table S1 Latin names of species listed in the manuscript and a summary of the number of animals returned dead and alive during the study.** The majority of animals returned dead were retained for positive identification; those released alive were identified by householders, so identifications may not always have been accurate.

| **Taxon** | **Species** | **Number returned dead** | **Number returned alive** | **Total** |
| --- | --- | --- | --- | --- |
| Mammals | Wood mouse *Apodemus sylvaticus* | 421 | 39 | 460 |
|  | Bank vole *Myodes glareolus* | 28 | 1 | 29 |
|  | Field vole *Microtus agrestis* | 2 | 0 | 2 |
|  | Brown rat *Rattus norvegicus* | 58 | 1 | 59 |
|  | Common shrew *Sorex araneus* | 27 | 1 | 28 |
|  | Pygmy shrew *Sorex minutus* | 4 | 0 | 4 |
|  | Water shrew *Neomys fodiens* | 2 | 0 | 2 |
|  | Unidentified small mammal^1^ | 102 | 47 | 149 |
|  | Grey squirrel *Sciurus carolinensis* | 3 | 0 | 3 |
|  | Pipistrelle bat *Pipistrellus* sp. | 4 | 1 | 5 |
|  | Mole *Talpa europaea* | 1 | 0 | 1 |
|  | Weasel *Mustela nivalis* | 1 | 0 | 1 |
|  | ***Subtotal*** | ***653*** | ***90*** | ***743*** |
| Birds | Robin *Erithacus rubecula* | 48 | 2 | 50 |
|  | Dunnock *Prunella modularis* | 16 | 1 | 17 |
|  | Blackbird *Turdus merula* | 48 | 11 | 59 |
|  | Great tit *Parus major* | 20 | 2 | 22 |
|  | Blue tit *Parus caeruleus* | 30 | 3 | 33 |
|  | Starling *Sturnus vulgaris* | 5 | 1 | 6 |
|  | Song thrush *Turdus philomelos* | 1 | 2 | 3 |
|  | House sparrow *Passer domesticus* | 9 | 3 | 12 |
|  | Chaffinch *Fringilla coelebs* | 7 | 0 | 7 |
|  | Long-tailed tit *Aegithalos caudatus* | 3 | 2 | 5 |
|  | Magpie *Pica pica* | 1 | 1 | 2 |
|  | Wren *Troglodytes troglodytes* | 7 | 1 | 8 |
|  | Goldcrest *Regulus regulus* | 1 | 0 | 1 |
|  | Greenfinch *Carduelis chloris* | 3 | 1 | 4 |
|  | Blackcap *Sylvia atricapilla* | 2 | 2 | 4 |
|  | Nuthatch *Sitta europaea* | 1 | 0 | 1 |
|  | Unidentified small bird^2^ | 52 | 6 | 58 |
|  | Mallard *Anas platyrhynchos*^3^ | 12 | 0 | 12 |
|  | Wood pigeon *Columba palumbus* | 33 | 2 | 35 |
|  | Collared dove *Streptopelia decaocto* | 5 | 1 | 6 |
|  | Unidentified^4^ | 2 | 1 | 3 |
|  | Moorhen *Gallinula chloropus*^3^ | 1 | 0 | 1 |
|  | Great spotted woodpecker *Dendrocopos major* | 1 | 0 | 1 |
|  | ***Subtotal*** | ***308*** | ***42*** | ***350*** |
| Bird eggs |  | 0 | 1 | 1 |
| Amphibians | Common frog *Rana temporaria* | 27 | 23 | 50 |
| Reptiles | Slow worm *Anguis fragilis* | 0 | 4 | 4 |
|  | Grass snake *Natrix natrix* | 0 | 2 | 2 |
| **TOTAL** |  | **988** | **162** | **1150** |

^1^ Individuals recorded as e.g. “mouse”. ^2^ Individuals recorded as e.g. “small bird”. ^3^ Relates to predation on ducklings rather than adult birds. ^4^ Individuals recorded as “pigeon”
